# From Burning Problem to Growing Value: Development of Sustainable Mushroom Substrates for Cost Reduction and Yield Enhancement

**DOI:** 10.3390/jof12090661

**Published:** 2026-09-02

**Authors:** Orlavanh Xayyavong, Phongeun Sysouphanthong, Kritsana Jatuwong, Saisamorn Lumyong, Worawoot Aiduang

**Affiliations:** 1 Doctor of Philosophy Program in Applied Microbiology (International Program), Faculty of Science, Chiang Mai University, Chiang Mai 50200, Thailand; xorlavanh@yahoo.com; 2 Department of Biology, Faculty of Science, Champasack University, Pakse 16010, Laos; 3 Department of Biology, Faculty of Science, Chiang Mai University, Chiang Mai 50200, Thailand; psphongeun@gmail.com (P.S.); kritsana.ja@cmu.ac.th (K.J.); 4 Office of Research Administration, Chiang Mai University, Chiang Mai 50200, Thailand; 5 Center of Excellence in Microbial Diversity and Sustainable Utilization, Chiang Mai University, Chiang Mai 50200, Thailand

**Keywords:** biomass utilization, *Lentinus* mushrooms, oyster mushrooms, particulate matter 2.5, sustainable development goals 2 and 12

## Abstract

Open-field burning of agricultural residues is a persistent environmental challenge that contributes to greenhouse gas emissions, air pollution, and the loss of valuable biomass resources. This study developed sustainable mushroom cultivation substrates by replacing 50% of conventional sawdust with locally available agricultural residues, including corn stalks, rice straw, sugarcane leaves, and leaf litter, to reduce production costs, enhance mushroom productivity, and promote circular bioeconomy practices. The physicochemical properties, mycelial growth, contamination, yield performance, nutritional composition, economic feasibility, and environmental benefits of the alternative substrates were evaluated using *Lentinus sajor-caju* and *Pleurotus* species. Among the tested formulations, corn stalk-based substrates exhibited the most favorable characteristics, with improved nitrogen availability and a more balanced C/N ratio, resulting in faster colonization, lower contamination, quicker fruiting body formation period, and superior biological efficiency. The corn stalk formulation achieved the highest productivity across species, including biological efficiencies of 61.99% for *L. sajor-caju*, 102.79% for *P. cornucopiae*, 99.40% for *P. ostreatus*, and 101.96% for *P. pulmonarius*. In addition, alternative substrates maintained or enhanced mushroom nutritional quality, with high protein (18.22–31.37%), dietary fiber (up to 30.58%), and low-fat contents (1.11–1.95%). Economic analysis demonstrated that a 50:50 sawdust-biomass substitution strategy substantially reduced substrate costs, achieving approximately 30% savings at industrial production scales. Overall, this study demonstrates that converting agricultural residues into high-value mushroom substrates provides an effective strategy to improve production efficiency, reduce costs, and advance sustainable mushroom cultivation systems.

## 1. Introduction

The widespread practice of open biomass burning, particularly in agricultural and forestry areas, has emerged as a critical environmental and public health challenge [1]. Large quantities of agricultural residues, such as rice straw, corn residues, sugarcane leaves, and forest leaf litter, are often disposed of through burning due to minimal use strategies and the high cost of waste management [2]. This practice releases substantial amounts of particulate matter (PM), including fine particles with an aerodynamic diameter of ≤2.5 μm (PM_2.5_), which can penetrate deep into the respiratory system and pose significant health risks. Consequently, biomass burning contributes to severe air-quality deterioration and greenhouse gas emissions while simultaneously causing the loss of valuable organic resources that could potentially be transformed into economically beneficial products [3].

In recent years, increasing attention has been directed toward sustainable biomass valorization strategies that convert agricultural waste into high-value materials. Among these, mushroom cultivation has gained considerable interest as an environmentally friendly and economically viable approach [4]. Edible mushrooms, particularly species within the genera *Lentinus* and *Pleurotus*, are widely recognized for their high economic value and their ability to degrade lignocellulosic materials, converting low-value biomass into nutrient-rich food products [5,6]. This biological process not only reduces waste but also supports circular bioeconomy systems and contributes to the United Nations Sustainable Development Goals (SDGs), particularly SDG 2 (zero hunger) and SDG 12 (responsible consumption and production), by transforming agricultural residues into valuable food and agricultural inputs [4].

However, the efficiency of mushroom cultivation is highly dependent on the composition and quality of the substrate [7]. In the recent past, the industry has continued to rely heavily on conventional materials, particularly rubber wood sawdust, which is becoming increasingly scarce and costly in high-demand regions, thereby driving up production expenses. In response, the development of alternative substrate formulations using locally available biomass residues has become essential to reduce costs, improve yield performance, and support long-term sustainability [8,9]. Despite this potential, variations in the chemical composition of different biomass types, such as cellulose, hemicellulose, lignin content, and nutrient availability, can significantly affect mycelial growth, colonization efficiency, and fruiting body development, along with overall product quality [9,10,11].

This study aims to address these challenges by developing and evaluating sustainable substrate formulations derived from widespread agricultural and forestry residues, including corn stalk, rice straw, sugarcane leaves, together with a typical dipterocarp and teak leaf litter. These materials were combined with sawdust in equal proportions to investigate their suitability for mushroom cultivation. Previous studies have suggested that combining sawdust with alternative biomass sources, particularly at a 50:50 ratio, can improve the balance of physical and chemical properties within the substrate, thereby enhancing its performance and promoting optimal mycelial growth as well as overall mushroom yield [12,13,14,15]. The study comprehensively examines substrate chemical properties, mycelial growth rate and density, spawn colonization time, contamination rates, and overall yield productivity of *Lentinus* and *Pleurotus* species. These factors provide information regarding the relationships between substrate suitability, fungal colonization efficiency, cultivation quality, and overall production productivity. In addition, the nutritional composition of harvested mushrooms is analyzed to assess product quality, while economic feasibility assessments are conducted to determine the broader sustainability and practical benefits of the proposed approach.

Thus, the progress of this work lies not simply in testing alternative residues, but in integrating biological performance, substrate characteristics, nutritional quality, and economic feasibility to identify practical residue-based substrate formulations. Through integrating cultivation performance with economic considerations, this study proposes to transform the conventional burning problem into a value-generating opportunity. The findings are expected to contribute to the development of cost-effective and sustainable mushroom cultivation systems, supporting waste valorization, reducing environmental impacts, and enhancing the resilience of agricultural circles.

## 2. Materials and Methods

### 2.1. Collecting and Preparing Raw Substrate Materials

Five types of dried agricultural and forest biomass, including corn stalks, rice straw, sugarcane leaves, dipterocarp leaves, and teak leaf litter, were used as the primary substrates in this study. These biomass residues are among the major contributors to open burning practices in Thailand, particularly in the northern region [2]. The biomass was collected from corn, rice, and sugarcane fields, as well as from a local natural forest around Chiang Mai University in northern Thailand. Prior to use, all substrates were processed using a mechanical chipper and subsequently sieved through a 10 mm mesh. Particles with sizes ranging from 1 to 10 mm were retained and used for further experimentation. Processing losses during chipping and sieving were recorded as approximately 20–25% of the initial dry biomass, and the retained fraction was used for substrate preparation.

Rubber tree sawdust was selected and used as the primary substrate control due to its widespread application in commercial mushroom cultivation. The sawdust was sourced from a local sawmill in northern Thailand and sieved prior to use to remove residual materials and obtain a uniform particle ≤2 mm in size.

### 2.2. Source of Fungal Species and Preparation of Mycelial Inoculum

Pure cultures of four edible mushroom species were obtained from the culture collections of the Thailand Bioresource Research Center (TBRC), Pathum Thani, and the Sustainable Development of Biological Resources Laboratory (SDBR Lab), Faculty of Science, Chiang Mai University, Chiang Mai, Thailand. The selected species comprised one *Lentinus* species (*Lentinus sajor-caju* TBRC 6266) and three oyster mushrooms, namely *Pleurotus cornucopiae* CMU-NK1270, *Pleurotus ostreatus* CMU-NK1071, and *Pleurotus pulmonarius* CMU-NK1348. These genera represent some of the most economically important cultivated mushrooms in Thailand. Prior to experimentation, each fungal strain was sub-cultured on potato dextrose agar (PDA; Difco, BBL™, Sparks, MD, USA) plates and incubated at 30 °C for 7 days to provide active mycelial growth for the production of the inoculum and subsequent experiments [16].

For inoculum preparation, sorghum grains were selected as a nutrient-rich substrate. The grains were thoroughly washed and boiled for 20 min. After boiling, excess water was carefully drained to prevent moisture accumulation during sterilization. Approximately 150 g of the prepared grains were then transferred into glass bottles, sealed with cotton plugs, and sterilized by autoclaving at 121 °C for 20 min. Following cooling to room temperature, each bottle was aseptically inoculated with five mycelial plugs (5 mm in diameter) obtained from actively growing PDA cultures of the respective mushroom species, with 15 bottles prepared per species. The inoculated bottles were subsequently incubated at 30 °C for two weeks under dark conditions, allowing complete mycelial colonization of the sorghum substrate [17].

### 2.3. Formulation of Substrate Mixtures and Analysis of Chemical Properties

Each substrate formulation was prepared by combining sawdust with individual agricultural or forest biomass materials in equal proportions (50:50, *w*/*w*), while 100% sawdust served as the control treatment. This ratio has been widely recommended, as blending sawdust with alternative biomass sources at a 50:50 proportion helps optimize the physical and chemical characteristics of the substrate, thereby enhancing mycelial growth and improving mushroom yield [12,13,14,15]. All substrate mixtures were supplemented with additional ingredients, including 5% rice bran, 1% calcium carbonate (CaCO_3_), 2% calcium sulfate (CaSO_4_), and 0.2% sodium sulfate (Na_2_SO_4_) [18], following the formulations presented in Table 1. The prepared substrates were then oven-dried at 60 °C for 24 h and subsequently ground to a fine particle size (<2 mm) prior to chemical analysis. Notably, this drying and grinding process was performed only for chemical analysis; the substrates used for spawn production retained their natural dryness and particle size.

The pH of each substrate was determined using a 1:10 (*w*/*v*) substrate-to-distilled water suspension and measured using a Sartorius PB-10 pH meter (Sartorius, Göttingen, Germany). Organic carbon (OC) content was analyzed following the Walkley and Black method (1934), and organic matter (OM) was calculated based on OC using a conversion factor of 1.724 (OM = OC × 1.724). Total nitrogen (N) content was determined using the Kjeldahl method, and the carbon-to-nitrogen (C/N) ratio was subsequently calculated. All analyses were conducted at the Agricultural Technology Services Center, Faculty of Agriculture, Chiang Mai University, Chiang Mai, Thailand, with each treatment performed in triplicate.

### 2.4. Preparation of Mushroom Spawn for Cultivation

#### 2.4.1. Producing *Lentinus* Spawn

The naturally dried substrate formulations described in Section 2.3, without oven-drying, were used for spawn production. Each formulation was adjusted to a final moisture content of approximately 60–70% prior to use. A total of 500 g of the prepared substrate was packed into polypropylene (PP) bags (3.5 × 11.5 inches), fitted with a PP ring, and sealed with a cotton-plastic plug. The bags were sterilized at 121 °C for 1 h and allowed to cool at room temperature for 24 h. Subsequently, each bag was inoculated with 5 g of prepared mycelial inoculum under clean conditions. The inoculated bags were incubated in a dark room at ambient temperature (25–33 °C) until complete mycelial colonization was achieved [17]. A total of thirty replicates were prepared for each treatment.

#### 2.4.2. Producing *Pleurotus* Spawn

The cultivation of three *Pleurotus* (oyster mushroom) species was conducted under farm-scale conditions to evaluate practical applicability. Substrate formulations described in Section 2.3 (without oven-drying) were first adjusted to a moisture content of approximately 60–70% to provide optimal conditions for mycelial growth. Approximately 500 g of the prepared substrate was then filled into 3.5 × 11.5-inch PP bags, each fitted with a PP neck ring and plugged with a PP rod stopper. The packed bags were placed in tunnels and subjected to steam pasteurization at 75–100 °C for 10 h to provide effective disinfection [5]. Subsequently, the bags were allowed to cool at ambient temperature for 24 h. After cooling, each bag was carefully inoculated with approximately 5 g of prepared mycelial inoculum under clean conditions [17]. The inoculated bags were then incubated in darkness under typical farmhouse conditions (24–36 °C) until full mycelial colonization was achieved. Each substrate treatment consisted of fifty replicates.

### 2.5. Evaluation of Mycelial Growth on Spawn, Contamination Rates, and Fruiting Body Induction

#### 2.5.1. Mycelial Growth Performance and Contamination Rates in *Lentinus* Spawn

Mycelial growth performance was evaluated by recording the time required for full substrate colonization, along with the contamination rate. Vegetative growth was monitored through direct observation, while the presence of contaminants, particularly green mold from *Trichoderma* spp. and other visible genera, was carefully documented [19]. After full colonization, the bags were transferred to a small-scale greenhouse and arranged horizontally on cultivation shelves. To induce fruiting development, the PP ring was removed on the first day, and the bag opening was gently loosened to expose the colonized substrate. On the second day, a slit was made at the neck of each bag, and the plastic covering was partially removed to stimulate fruiting body development. Moisture management commenced on the third day, with regular watering to maintain optimal humidity. The time to primordia formation and the appearance of the first harvestable fruiting bodies were recorded to evaluate cultivation performance [18,19,20].

#### 2.5.2. Mycelial Growth Performance and Contamination Rates in *Pleurotus* Spawn

The mycelial growth performance of three oyster mushroom strains was evaluated by measuring the time required for complete substrate colonization, along with the corresponding contamination rates, following the technique described in Section 2.5.1. Upon full colonization, the spawn bags were transferred to a local mushroom cultivation house in Chiang Mai Province, Thailand, for fruiting body development. At this stage, the cotton plugs were removed to facilitate fruiting. Environmental conditions in the mushroom house were maintained at temperatures ranging from approximately 25.5 °C to 33.5 °C, with relative humidity levels between 75% and 80%. Moisture was carefully managed through daily watering to sustain suitable humidity for fruiting bodies [21]. The duration to primordia initiation and the appearance of the first harvestable fruiting bodies were recorded as key indicators of cultivation performance.

### 2.6. Mushroom Harvesting and Yield Performance Evaluation

#### 2.6.1. Harvesting and Yield of *Lentinus* Mushroom

For mushroom production in small-scale greenhouse setups, 22 fully colonized and uncontaminated bags for each substrate formulation were selected and continuously monitored for yield assessment over a period of approximately 12 weeks. Bags showing contamination were excluded from the yield assessment and were not included in the subsequent statistical analysis. During the experimental period (June–September 2025), the study was conducted in the rainy season, when ambient humidity was naturally high. Greenhouse relative humidity was maintained at approximately 75–85% with watering once daily, and temperatures were around 25 to 34 °C. Mushrooms were harvested after the maturity of fruiting bodies. The market quality of the harvested basidiocarps was assessed according to the criteria described by Chiejina and Osibe [20]. Each collected mushroom was weighed and numbered, with useful fruiting bodies typically exhibiting cap diameters of approximately 3–7 cm across treatments. Freshly harvested samples were subsequently dried in a hot-air oven at 60 °C for 24–48 h until a constant weight was obtained, after which dry weights were recorded. Total mushroom weight per bag and the number of flushes were documented for each treatment over the entire cultivation period. Furthermore, biological efficiency (BE) was determined to assess production performance using the following equation: BE (%) = [Fresh weight of harvested mushrooms (g)/Dry weight of substrate (g)] × 100.

#### 2.6.2. Harvesting and Yield of *Pleurotus* Mushroom

Under farm-scale cultivation conditions in the mushroom house, 20 fully colonized and uncontaminated substrate bags for each treatment were selected and measured for yield evaluation during the duration of about 12 weeks. Contaminated bags were withdrawn from the production trial and excluded from yield measurements and statistical analyses. The study was conducted between June and September 2025, during the rainy season, when environmental humidity levels were naturally high. The relative humidity within the mushroom house was maintained at approximately 75–80% through daily watering practices, while temperatures ranged from 25 to 34 °C. Fruiting bodies were harvested at full maturity, and quality was assessed according to Ref. [22] for *Pleurotus* mushrooms. After harvesting, the mushrooms were placed in plastic containers lined with moist paper to preserve freshness and subsequently transported to the laboratory. All samples were counted and initially weighed to determine fresh biomass. Marketable fruiting bodies generally exhibited cap diameters ranging from 3.6 to 7 cm across all treatments. Fresh samples were then oven-dried at 60 °C for 24–48 h until a stable weight was obtained. Total mushroom weight per bag and the number of flushes for each treatment were recorded over the cultivation period. Biological efficiency was also calculated as an indicator of production efficiency using the formula described in Section 2.6.1.

### 2.7. Nutritional Composition Analysis of Harvested Lentinus and Pleurotus Mushrooms

The nutritional composition of dried samples of both *Lentinus* and *Pleurotus* mushrooms that were collected from various substrate formulations was analyzed using proximate analytical methods. The major components examined included crude protein, crude fat, ash, and crude fiber, following standard methods established by the Association of Official Analytical Chemists (AOAC). Crude protein content was calculated using a nitrogen-to-protein conversion factor of 4.38. Total carbohydrate content was estimated by difference using the following equation: Total carbohydrates (%) = 100 − (% moisture + % crude protein + % crude fat + % crude fiber + % ash).

The energy value of each harvested *Lentinus* and *Pleurotus* mushroom sample was determined using bomb calorimetry in accordance with AOAC standard methods for food energy analysis. All analyses were conducted at the Central Laboratory and the Animal Nutrition Laboratory, Department of Animal and Aquatic Sciences, Faculty of Agriculture, Chiang Mai University, Thailand. Each sample was analyzed in triplicate.

### 2.8. Statistical Analysis and Economic Feasibility Assessment

Statistical analysis was performed using one-way analysis of variance (ANOVA) with SPSS software (version 17.0; SPSS Inc., Chicago, IL, USA). Differences among mean values were evaluated using Duncan’s multiple range test at a significance level of *p* < 0.05. Economic feasibility was assessed by comparing production costs with those of conventional practices.

## 3. Results and Discussions

### 3.1. Chemical Properties of Substrate Formulation

The chemical characterization of the mixed substrate formulations demonstrated significant differences among treatments, indicating that the incorporation of agricultural residues markedly altered the physicochemical properties of the cultivation substrates (Table 2). Substrate pH values ranged from 6.17 to 7.11, remaining within the slightly acidic to neutral range generally considered suitable for mushroom mycelial colonization, extracellular enzymatic activity, and lignocellulosic degradation in *Lentinus* and *Pleurotus* species, which generally thrive at pH 5.0–8.0 [23]. The control treatment (T1, 100% sawdust) exhibited the highest pH (7.11), whereas formulations containing teak leaf litter (T6) and dipterocarp leaf litter (T3) produced significantly lower pH values (6.17 and 6.21, respectively). This reduction in pH may be attributed to the release of dissolved organic acids and other acidic compounds from leaf-derived biomass during hydration and substrate preparation, which increased proton availability and consequently lowered the substrate pH [24].

Organic carbon and OM contents were highest in the sawdust-only substrate (T1), reaching 57.12% and 98.48%, respectively. Replacing half of the sawdust with biomass residues slightly reduced both parameters, reflecting differences in lignocellulosic composition and the relative proportions of cellulose, hemicellulose, lignin, and mineral constituents among the biomass sources. Nevertheless, all mixed substrates retained high OM contents (>84%), providing abundant carbon-rich materials required for fungal metabolism and biomass production.

The most pronounced variation was observed in total nitrogen content and the resulting C/N ratio. Corn stalk addition (T2) significantly increased nitrogen concentration to 0.55%, followed by teak leaf litter (T6; 0.48%) and dipterocarp leaf litter (T3; 0.43%), whereas the control (sawdust) contained only 0.22% nitrogen. Consequently, T2 and T6 exhibited substantially lower C/N ratios (96.93 and 111.88, respectively) compared with the extremely high ratio of the sawdust control (258.12). In contrast, sugarcane leaves (T5) and rice straw (T4) maintained relatively high C/N ratios of 194.23 and 162.33, respectively.

These findings demonstrate that blending sawdust with locally available agricultural residues effectively improved substrate nutritional balance by increasing nitrogen availability while reducing excessive carbon levels. Previous studies have consistently reported that substrate formulations with moderate C/N ratios promote more efficient lignocellulose degradation, accelerate mycelial colonization, and enhance nutrient utilization, ultimately leading to improved mushroom productivity [25,26,27]. Although sawdust remains an excellent structural substrate due to its high organic matter content, its extremely high C/N ratio may limit fungal growth because of insufficient nitrogen availability [9]. Conversely, corn stalks and teak leaf litter produced more balanced nutritional profiles that are likely to better support fungal decomposition and fruiting body development [27]. Therefore, the chemical properties observed in the mixed substrates may provide strong indications that biomass residues can serve as sustainable alternatives to conventional sawdust.

### 3.2. Mycelial Growth, Contamination, and Fruiting Body Induction in Spawn Bags

The performance of the different substrate formulations significantly influenced substrate colonization, contamination incidence, primordia initiation, and the production cycle of all four mushroom species (Table 3). Across all treatments, *L. sajor-caju* exhibited the fastest colonization and shortest cultivation period, whereas the *Pleurotus* species required relatively longer incubation and fruiting periods. Among the alternative substrates, the corn stalk formulation (A–D-T2) consistently produced the most favorable cultivation performance. It significantly shortened substrate colonization to 22.75, 25.20, 25.40, and 25.10 days for *L. sajor-caju*, *P. cornucopiae*, *P. ostreatus*, and *P. pulmonarius*, respectively, while also promoting the earliest primordia formation and first harvest. Fruiting bodies from T2 (A–D) were harvested within only 30.80–33.70 days after bag opening, representing the shortest production cycle among all treatments. In contrast, the rice straw formulation (A–D-T4) consistently delayed mycelial colonization (28.90–38.90 days), primordia formation (39.85–45.95 days), and first harvest (42.70–48.65 days), indicating potentially less than optimal substrate suitability.

**Figure 1:**
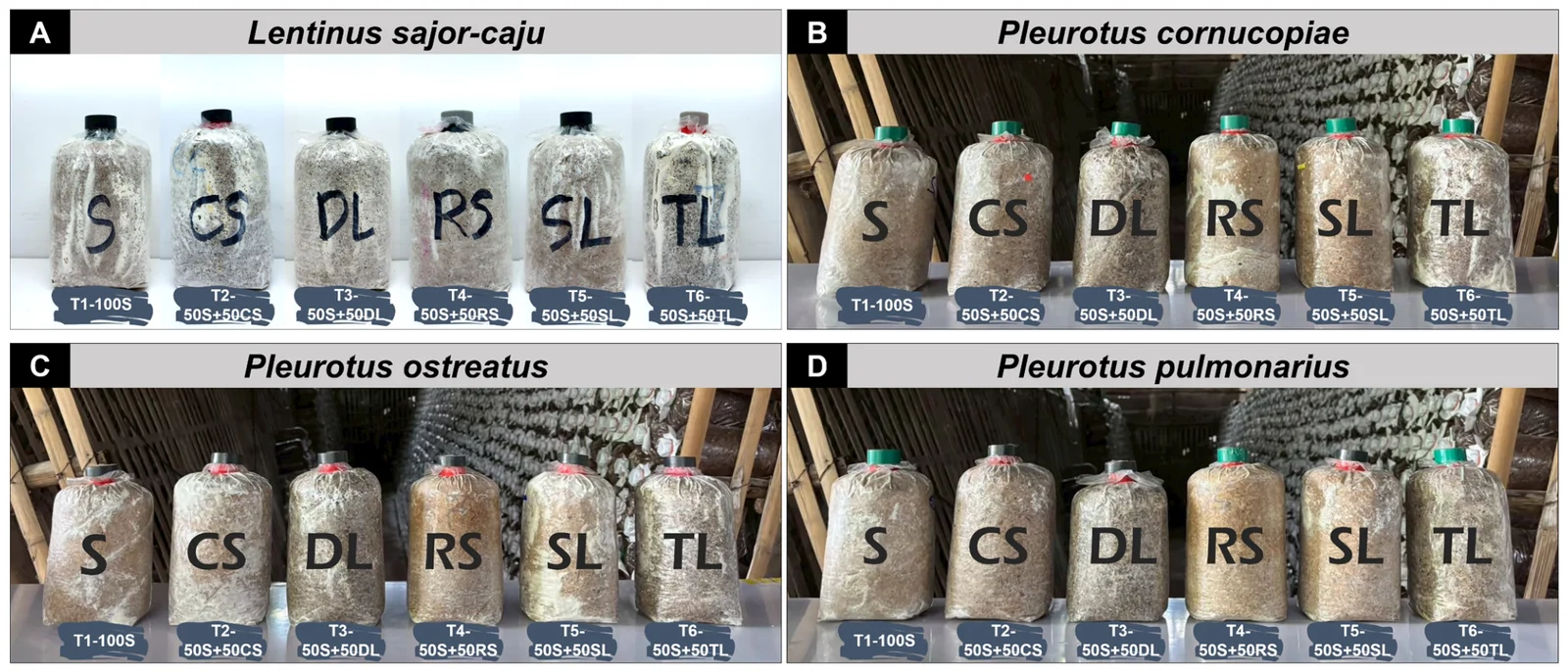
Mycelial colonization characteristics of different substrate formulations for each mushroom species, including *L. sajor-caju* (**A**), *P. cornucopiae* (**B**), *P. ostreatus* (**C**), and *P. pulmonarius* (**D**), observed under small-scale greenhouse conditions (**A**) and typical farmhouse conditions (**B**–**D**). Treatment formulations: T1-100S = 100% sawdust (control); T2-50S+50CS = 50% sawdust + 50% corn stalks; T3-50S+50DL = 50% sawdust + 50% dipterocarp leaf litter; T4-50S+50RS = 50% sawdust + 50% rice straw; T5-50S+50SL = 50% sawdust + 50% sugarcane leaves; T6-50S+50TL = 50% sawdust + 50% teak leaf litter.

Substrate formulation also markedly affected contamination during spawn running. Substrate prepared using corn stalk (T2), sugarcane leaves (T5), and teak leaf litter (T6) mostly maintained relatively low contamination levels, particularly for *L. sajor-caju*, where contamination was limited to only 3.3%. Conversely, rice straw exhibited the highest contamination rates across all mushroom species, reaching 16.7%, 46%, 56%, and 52% for *L. sajor-caju*, *P. cornucopiae*, *P. ostreatus*, and *P. pulmonarius*, respectively. These elevated contamination levels were accompanied by prolonged colonization, delayed fruiting, and mushroom deformation or decomposition, suggesting that microbial competition substantially reduced cultivation efficiency [28]. The spawn bags that remained completely free of contamination throughout the spawn-running period, with no visible indication of microbial infection, are presented in Figure 1. Contaminated bags were withdrawn from the yielding phase and excluded from subsequent yield measurements and statistical analyses because contamination prevented sufficient assessment of mushroom productivity. Accordingly, the contamination rates reported in Table 3 reflect the proportion of contaminated bags, whereas yield-related analyses were based only on uncontaminated bags that successfully completed the production cycle.

The superior performance of the corn stalk substrate can be attributed to its balanced physicochemical properties, including improved nitrogen availability and a more favorable C/N ratio, which likely enhanced extracellular enzyme production and accelerated lignocellulosic degradation [27]. Nevertheless, residue type and C/N ratio may act as interacting factors; therefore, future studies should standardize the C/N ratio across treatments to more clearly isolate the effects of substrate composition. Rapid substrate colonization, however, is particularly advantageous because it allows mushroom mycelia to occupy the substrate quickly, minimizing opportunities for contaminating microorganisms to establish and compete for nutrients [29,30]. In contrast, rice straw generally contains higher silica levels, a dense fibrous structure, and indigenous microbial populations that can slow mycelial penetration and increase contamination risk when pretreatment is insufficient [31,32,33]. Although sugarcane leaves and teak leaf litter did not outperform corn stalks, they produced cultivation performances comparable to the conventional sawdust substrate, demonstrating their potential as partial substitutes.

Overall, these findings indicate that the choice of agricultural residue affects not only mycelial growth but also the entire production cycle, from spawn running to harvest. This finding is consistent with Zakil et al. [14,15], who reported that a 50% sawdust + 50% agricultural residue mixture improved fresh mushroom yield, biological efficiency, and overall production performance. Among the formulations evaluated in the present study, replacing 50% of sawdust with corn stalks substantially improved cultivation efficiency by reducing contamination, accelerating substrate colonization, shortening the time to primordia formation, and enabling earlier harvesting. Therefore, converting crop residues that are commonly disposed of through open-field burning into mushroom cultivation substrates represents a practical circular bioeconomy strategy that reduces production costs, shortens cultivation time, improves farm productivity, and simultaneously mitigates environmental pollution caused by biomass burning.

### 3.3. Comparative Yield and Biological Efficiency in Various Substrate Formulations

The productivity of the four cultivated mushroom species varied significantly among substrate formulations, demonstrating that the incorporation of different agricultural residues markedly influenced yield variables and BE (Table 4). Overall, the mixed substrate containing 50% sawdust + 50% corn stalk (T2) consistently produced the highest productivity across all tested species.

**Figure 2:**
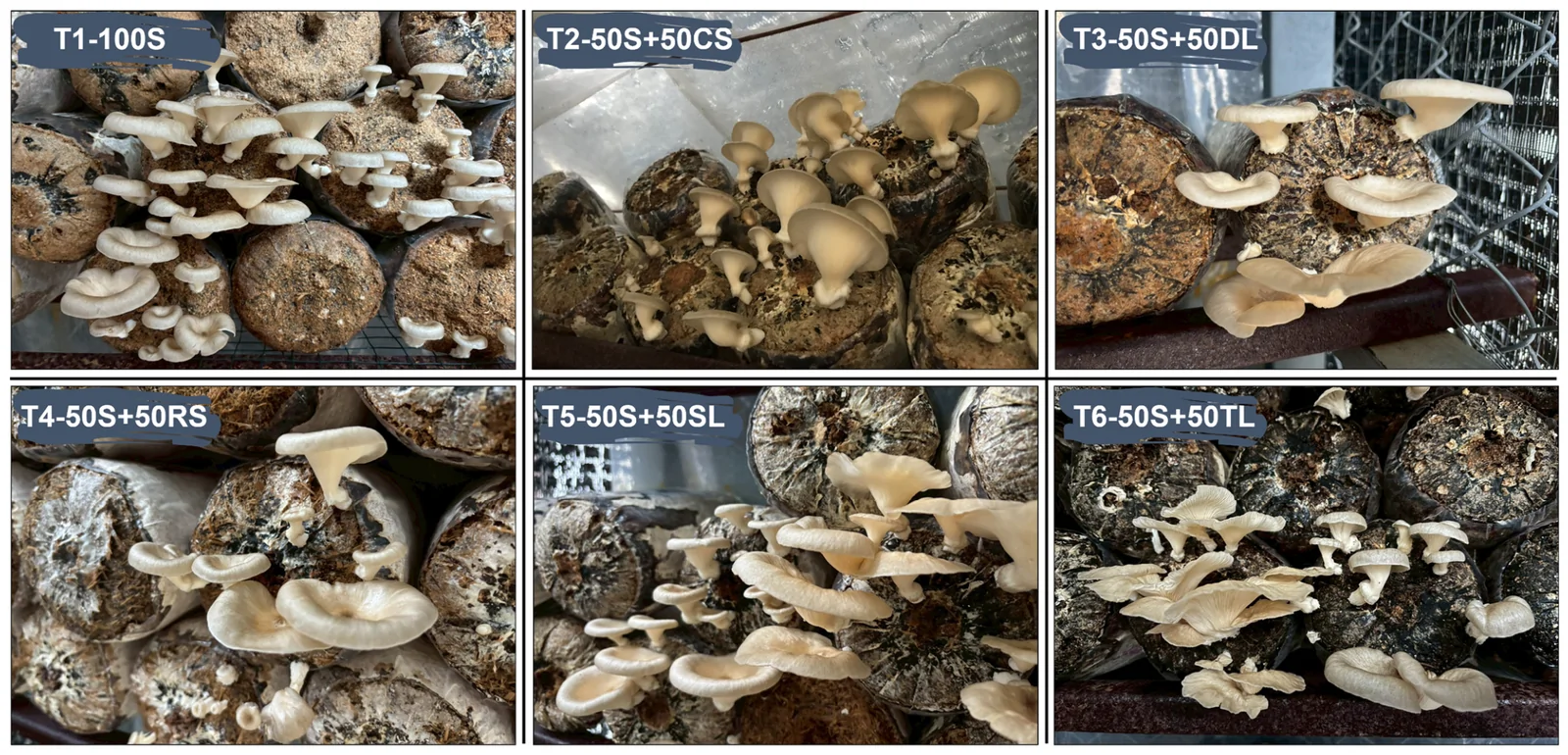
Development of *Lentinus* mushroom fruiting bodies observed across different substrate formulations in each treatment following bag opening. Treatment formulations: T1-100S = 100% sawdust (control); T2-50S+50CS = 50% sawdust + 50% corn stalks; T3-50S+50DL = 50% sawdust + 50% dipterocarp leaf litter; T4-50S+50RS = 50% sawdust + 50% rice straw; T5-50S+50SL = 50% sawdust + 50% sugarcane leaves; T6-50S+50TL = 50% sawdust + 50% teak leaf litter.

For *L. sajor-caju* (Figure 2), A-T2 resulted in the greatest fresh mushroom amount (70.75 g bag^−1^), highest dry weight (8.09 g bag^−1^), and superior BE (61.99%), significantly outperforming the conventional 100% sawdust substrate (A-T1). Comparable performance was also observed in the sugarcane leaf formulation (A-T5), whereas durian leaves (A-T3) and rice straw (A-T4) produced significantly fewer flushes, lower fruiting body numbers, and reduced BE.

**Figure 3:**
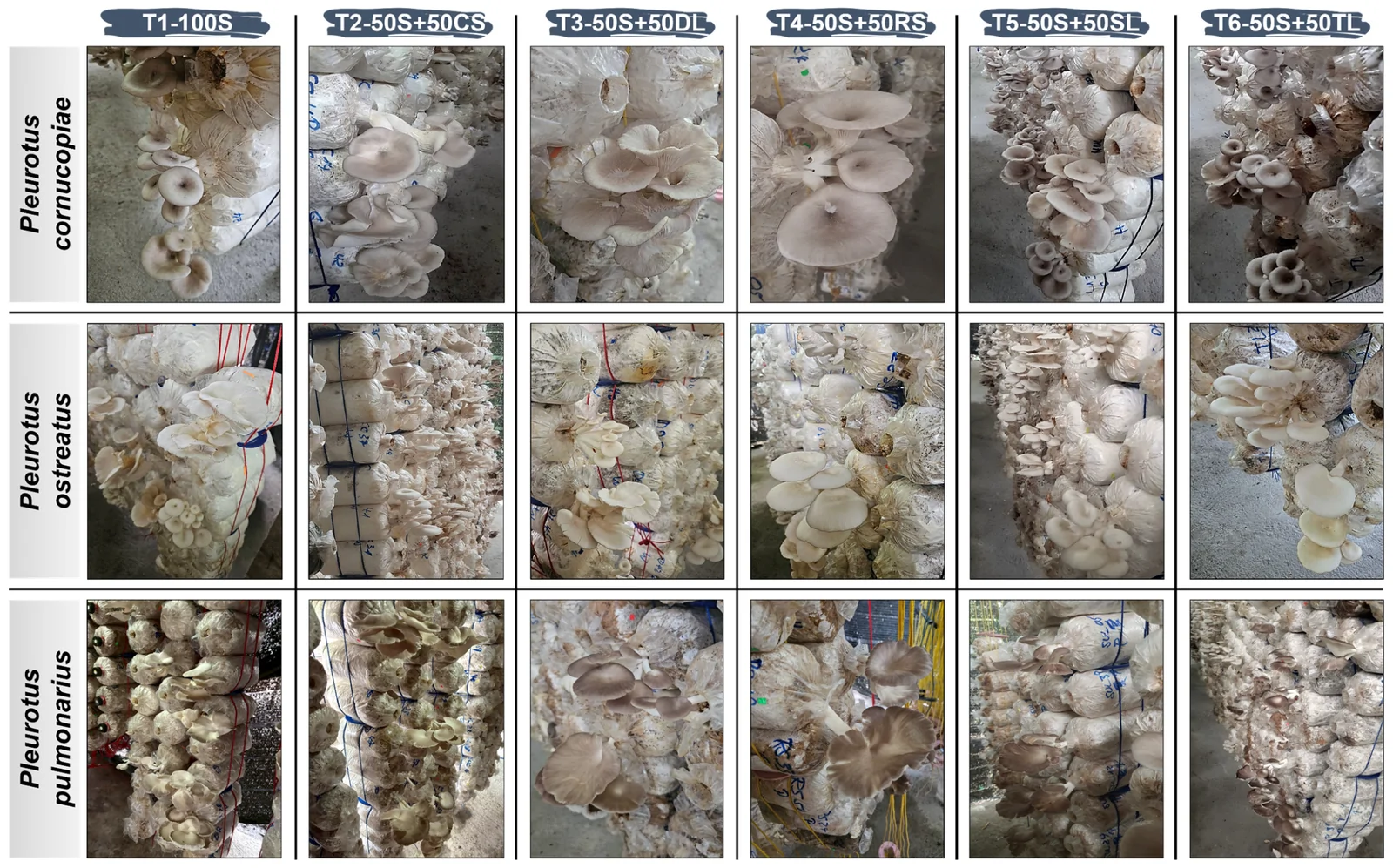
Development of *Pleurotus* fruiting bodies across different substrate formulations after fruiting induction. Treatment formulations: T1-100S = 100% sawdust (control); T2-50S+50CS = 50% sawdust + 50% corn stalks; T3-50S+50DL = 50% sawdust + 50% dipterocarp leaf litter; T4-50S+50RS = 50% sawdust + 50% rice straw; T5-50S+50SL = 50% sawdust + 50% sugarcane leaves; T6-50S+50TL = 50% sawdust + 50% teak leaf litter.

A similar trend was observed for *Pleurotus* species (Figure 3). For *P. cornucopiae*, B-T2 achieved the highest BE (102.79%) while maintaining fresh mushroom amounts comparable to B-T1, indicating more efficient substrate conversion into mushroom biomass. The sugarcane leaf (B-T5) and teak leaf (B-T6) substrates also supported relatively high production, whereas rice straw (B-T4) exhibited the poorest performance with the fewest flushes, lowest fruiting body production, and minimum BE. For *P. ostreatus*, C-T5 produced the highest fresh weight (97.43 g bag^−1^), although C-T2 achieved the greatest BE (99.40%), suggesting that corn stalk incorporation enhanced substrate utilization efficiency over simple mushroom amount improvement. In contrast, C-T4 and C-T6 significantly reduced productivity, highlighting the limited suitability of these substrates without further optimization. Among all species, *P. pulmonarius* exhibited the greatest production potential, with D-T2 generating the highest fresh mushroom amount (106.75 g bag^−1^) and BE (101.96%), followed closely by D-T5 (97.81%). These results indicate that corn stalk and sugarcane leaf provided a more favorable nutritional balance for rapid mycelial colonization and sustained fruiting than the conventional sawdust substrate.

Overall, the results clearly demonstrate that replacing half of rubber tree sawdust, a commonly used substrate for *Pleurotus* cultivation in Thailand [34], with corn stalk residues can substantially improve mushroom productivity and substrate conversion efficiency across multiple edible mushroom species. The improved performance of corn stalk-based substrates may reflect a combination of their nutritional composition, C/N ratio, physical structure, moisture retention, and aeration characteristics, which can collectively influence mycelial colonization and efficient lignocellulose degradation [35]. These findings agree with previous studies showing that biomass residues with favorable structural and nutritional characteristics can enhance enzymatic activity, nutrient availability, and fruiting performance [5,9,36,37]. Moreover, comparable findings have been reported in previous studies, where corn stalk residues effectively replaced hardwood sawdust for cultivating *Pleurotus* species while maintaining or improving BE [38,39,40]. In contrast, substrates containing dipterocarp leaf or rice straw generally exhibited lower biological efficiencies, probably because of their higher lignin content, silica accumulation, or inhibitory phenolic compounds that restrict mycelial growth and nutrient accessibility [30,41]. Collectively, these findings demonstrate that the incorporation of corn stalks, followed by sugarcane leaves, as partial substitutes for sawdust represents an effective and sustainable strategy for valorizing agricultural residues that are otherwise disposed of through open-field burning. This approach simultaneously lowers substrate production costs, improves BE, and supports the transition toward greener mushroom farming practices.

### 3.4. Proximate and Nutritional Properties of Mushrooms

The proximate and nutritional composition of the harvested mushrooms varied among species and substrate formulations, indicating that partial substitution of sawdust with agricultural residues influenced fruiting body quality while maintaining favorable nutritional characteristics (Table 5). Across all treatments, the dry matter content of the oven-dried mushroom samples remained relatively consistent, ranging from 90.27 to 90.97% in *L. sajor-caju*, 92.17–94.15% in *P. cornucopiae*, 92.99–94.78% in *P. ostreatus*, and 93.98–94.63% in *P. pulmonarius*. These values correspond to moisture contents of approximately 5.22–9.73% on a dry-matter basis. Thus, the alternative substrate formulations did not adversely affect post-harvest dry matter accumulation, which is an important quality parameter for storage stability and processing.

The ash content ranged from 4.20 to 4.78% in *L. sajor-caju*, 5.50–8.26% in *P. cornucopiae*, 6.26–7.42% in *P. ostreatus*, and 5.56–6.95% in *P. pulmonarius*, reflecting species-specific mineral accumulation capacities and differences in substrate mineral availability. Carbohydrates represented the major nutritional component, accounting for 35.44–37.70%, 45.14–57.54%, 48.35–57.80%, and 56.19–60.24% in the respective species. The highest carbohydrate content was observed in *P. pulmonarius* cultivated on the rice straw-based substrate (T4), suggesting that this agricultural residue effectively supported carbohydrate biosynthesis and structural polysaccharide formation.

Protein concentrations exhibited notable variation among treatments, ranging from 18.22 to 21.63% in *L. sajor-caju*, 20.72–31.37% in *P. cornucopiae*, 18.61–30.53% in *P. ostreatus*, and 22.86–25.43% in *P. pulmonarius*. Particularly, *P. cornucopiae* grown on the sawdust–dry leaf mixture (T3) produced the highest protein content (31.37%), followed by *P. ostreatus* under the same treatment (30.53%), indicating that dry leaf residues may enhance nitrogen utilization and protein biosynthesis during mushroom development. In contrast, lipid content remained consistently low (1.11–1.95%) across all species and treatments, confirming the inherently low-fat nature of edible mushrooms. Dietary fiber also remained relatively abundant, especially in *L. sajor-caju* (26.90–30.58%) and *P. ostreatus* cultivated on tea leaf residue (24.47%), highlighting their potential as valuable sources of functional dietary fiber.

Compared with values reported in previous studies, the nutritional profiles obtained in this study were generally within the ranges previously reported for cultivated *L. sajor-caju* and *Pleurotus* species, although some substrate-dependent differences were evident [21,42,43]. Previous studies have similarly reported substantial variation in protein, carbohydrate, ash, and fiber contents among *Pleurotus* species and cultivation substrates, supporting the influence of both fungal genotype and substrate composition on nutritional quality. Notably, the protein and carbohydrate contents observed in *P. cornucopiae* (20.7–31.4% and 45.1–57.5%, respectively) and *P. ostreatus* (18.6–30.5% and 48.3–57.8%, respectively) were comparable to, and in several cases higher than, values previously reported for *P. cornucopiae* (3.0–32.0% protein and 4.2–35.0% carbohydrate) and *P. ostreatus* (3.4–32.0% protein and 5.1–50.9% carbohydrate) [21,42,44], whereas the low lipid content observed across all treatments remained consistent with published data for edible mushrooms [42,44]. These comparisons indicate that biomass residues can produce nutritionally competitive mushrooms without compromising overall quality.

The calculated energy values varied from 380.81 to 405.00 kcal/100 g dry weight, reflecting differences in carbohydrate and protein composition rather than fat content. Overall, these findings demonstrate that substituting conventional sawdust with locally available agricultural residues did not compromise the nutritional quality of cultivated mushrooms. Instead, several alternative substrates enhanced desirable nutritional characteristics, particularly protein, fiber, and mineral contents. These improvements are likely associated with differences in lignocellulosic composition, nitrogen availability, and mineral profiles of the substrates, which directly influence fungal metabolism and nutrient translocation into developing fruiting bodies [5,45]. The results agree with previous studies reporting that substrate composition is a major determinant of mushroom nutritional quality, and they further demonstrate that recycling biomass residues not only reduces production costs and open-field burning but also generates high-value mushrooms with improved nutritional properties [27,46,47,48], thereby supporting environmentally sustainable and circular mushroom production systems.

### 3.5. Cost Analysis and Production Cost Reduction

The cost estimation and analysis revealed clear economic advantages of integrating agricultural biomass as a partial replacement for sawdust in mushroom substrate formulations across all production scales (Table 6). Farm size categories were classified according to Xayyavong et al. [49]. Under the conventional 100% sawdust system, substrate cost increased proportionally with production scale, ranging from USD 50–1400 for small-scale farms to more than USD 28,008 for industrial-scale operations. This indicates that raw material dependency on sawdust significantly drives production expenditure, especially at higher output levels.

The application of a 50:50 sawdust–biomass substitution strategy was estimated to consistently reduce sawdust demand across all farm scales. For small-scale farms, replacing 50% of the sawdust with biomass residues reduced sawdust consumption from 0.6 to 16.8 to 0.3–8.4 tons, decreasing sawdust costs from USD 50–1400 to USD 25–700. Although the additional biomass cost was relatively low (USD 10–280), the total substrate cost remained only USD 35–980, representing a substantial cost reduction compared with the use of 100% sawdust. Similarly, medium-scale operations showed a substantial reduction in sawdust expenditure from USD 1408–9333 to 704–4667. Although biomass input increased costs slightly (USD 283–1867), the overall substrate cost remained significantly lower than full sawdust systems at USD 987–6533. For large-scale farms, cost savings became even more pronounced, with total substrate costs decreasing to USD 6541–19,600 compared to over USD 9342–28,000 under conventional formulation. This trend demonstrates that cost reduction benefits scale positively with production volume, making biomass substitution increasingly attractive for commercial operations. At the industrial scale (>600,000 bags/year), potential savings were substantial, with total substrate costs reduced from above USD 28,008 to approximately USD 19,608, or a 30% reduction in value. This suggests that even one-half substitution ratios may offer significant financial relief in high-volume production systems.

From a sustainability perspective, the substitution strategy also reduces dependence on sawdust, a resource that is becoming increasingly scarce and costly. Agricultural biomass, often considered waste, is effectively valorized into a productive input, aligning with circular economy principles. Overall, the findings confirm that the 50:50 substrate formulation not only reduces production costs but also enhances resource efficiency across all farm scales. These results highlight a strong economic incentive for adopting alternative substrate materials in modern mushroom production systems in Thailand. Furthermore, the integration of biomass contributes to stabilizing long-term production costs by reducing susceptibility to sawdust price fluctuations. In conclusion, substrate innovation through partial biomass replacement represents a practical and scalable strategy for improving profitability and sustainability in mushroom cultivation systems.

## 4. Conclusions

- Agricultural residues effectively improved the physicochemical properties of mushroom substrates by enhancing nitrogen availability and balancing the C/N ratio, providing a more suitable environment for mycelial growth and fruiting development.
- Replacing 50% of sawdust with biomass residues, particularly corn stalks and sugarcane leaves, promoted overall faster mycelial colonization, reduced contamination risks, facilitated fruiting body formation, and enhanced mushroom productivity across *L. sajor-caju*, *P. cornucopiae*, *P. ostreatus*, and *P. pulmonarius*.
- Corn stalk-based substrates demonstrated the highest overall cultivation performance, achieving superior biological efficiency and mushroom yield, while alternative residues maintained favorable nutritional quality with high protein, carbohydrate, and dietary fiber contents.
- The 50:50 sawdust–biomass substrate strategy provided substantial economic benefits by reducing dependence on costly sawdust and lowering substrate production costs, with increasing advantages observed at larger production scales.
- Valorizing agricultural residues as mushroom substrates offers an effective circular bioeconomy approach by transforming biomass waste into high-value food products.
- In summary, this study demonstrates that integrating locally available agricultural residues into mushroom cultivation systems can simultaneously enhance production efficiency, reduce costs, and provide a practical solution for converting burning problems into sustainable agricultural value.
- Future studies should optimize residue-specific formulations and C/N ratios under comparable nutrient conditions, next to farm-based acceptance to confirm productivity, economic feasibility, and cultivation stability under commercial conditions.

## Figures and Tables

**Table 1:** Formulation of mushroom cultivation substrates based on the combination of sawdust and various biomass residues.

Treatment Code	Rubber Sawdust Proportion (%)	Biomass Residue Proportion (%)	Remarks
T1-100S	100	0	100% sawdust
T2-50S+50CS	50	50	50% sawdust + 50% corn stalks
T3-50S+50DL	50	50	50% sawdust + 50% dipterocarp leaf litter
T4-50S+50RS	50	50	50% sawdust + 50% rice straw
T5-50S+50SL	50	50	50% sawdust + 50% sugarcane leaves
T6-50S+50TL	50	50	50% sawdust + 50% teak leaf litter

**Note:** The formulations were adapted and improved from [14,15].

**Table 2:** Chemical properties of the mixed substrate formulations used for each treatment in this study.

Treatment	pH	OC (%)	OM (%)	N (%)	C/N
T1-100S	7.11 ± 0.10 ^a^	57.12 ± 0.73 ^a^	98.48 ± 1.26 ^a^	0.22 ± 0.03 ^e^	258.12 ± 31.36 ^a^
T2-50S+50CS	6.60 ± 0.44 ^b^	52.99 ± 0.43 ^c^	91.34 ± 0.74 ^c^	0.55 ± 0.01 ^a^	96.93 ± 1.61 ^d^
T3-50S+50DL	6.21 ± 0.08 ^c^	51.86 ± 0.71 ^d^	89.40 ± 1.23 ^d^	0.43 ± 0.01 ^c^	121.55 ± 2.04 ^d^
T4-50S+50RS	6.77 ± 0.03 ^ab^	49.18 ± 0.49 ^e^	84.79 ± 0.84 ^e^	0.30 ± 0.01 ^d^	162.33 ± 7.84 ^c^
T5-50S+50SL	6.75 ± 0.07 ^b^	54.34 ± 0.31 ^b^	93.69 ± 0.54 ^b^	0.28 ± 0.01 ^d^	194.23 ± 5.93 ^b^
T6-50S+50TL	6.17 ± 0.01 ^c^	53.31 ± 0.31 ^c^	91.91 ± 0.54 ^c^	0.48 ± 0.01 ^b^	111.88 ± 2.06 ^d^

**Note:** Different letters within the same column indicate statistically significant differences, as determined by Duncan’s multiple range test (*p* < 0.05) compared with the control. pH, potential of hydrogen; OC, organic carbon; OM, organic matter; N, total nitrogen; C/N, carbon-to-nitrogen ratio.

**Table 3:** Mycelial colonization of substrates, contamination rates in mushroom spawn, duration of primordia formation, and time to first harvest after bag opening.

Mushroom Species	Treatment	Contamination Rate (%)	Completed Substrate Colonization (Days)	Primordia Formation Period (Days)	First Harvestable Fruiting Bodies (Days)
*L. sajor-caju*	A-T1-100S	6.7	23.55 ± 2.37 ^bc^	32.20 ± 11.60 ^b^	33.95 ± 11.68 ^bc^
	A-T2-50S+50CS	3.3	22.75 ± 1.33 ^bc^	29.30 ± 8.65 ^b^	30.80 ± 8.52 ^c^
	A-T3-50S+50DL	3.3	24.15 ± 3.22 ^b^	39.50 ± 9.35 ^a^	40.10 ± 9.27 ^ab^
	A-T4-50S+50RS	16.7	28.90 ± 1.62 ^a^	41.35 ± 9.80 ^a^	42.70 ± 9.88 ^a^
	A-T5-50S+50SL	3.3	22.90 ± 1.89 ^bc^	32.05 ± 11.86 ^b^	33.60 ± 11.72 ^bc^
	A-T6-50S+50TL	3.3	22.65 ± 1.42 ^c^	36.25 ± 13.24 ^ab^	37.80 ± 12.89 ^abc^
*P. cornucopiae*	B-T1-100S	14	27.10 ± 3.46 ^bc^	32.10 ± 3.82 ^bc^	34.50 ± 3.95 ^bc^
	B-T2-50S+50CS	16	25.20 ± 2.38 ^d^	30.20 ± 3.17 ^c^	32.70 ± 3.48 ^c^
	B-T3-50S+50DL	30	25.95 ± 1.39 ^cd^	32.85 ± 2.03 ^b^	35.80 ± 2.46 ^b^
	B-T4-50S+50RS	46	31.85 ± 2.76 ^a^	39.85 ± 4.37 ^a^	42.90 ± 5.01 ^a^
	B-T5-50S+50SL	4	27.70 ± 1.98 ^b^	33.35 ± 2.21 ^b^	35.80 ± 2.44 ^b^
	B-T6-50S+50TL	22	25.85 ± 1.35 ^cd^	33.00 ± 3.11 ^b^	35.75 ± 3.68 ^b^
*P. ostreatus*	C-T1-100S	22	26.25 ± 1.77 ^c^	34.35 ± 4.22 ^b^	37.20 ± 4.85 ^b^
	C-T2-50S+50CS	14	25.40 ± 1.05 ^c^	29.70 ± 2.41 ^c^	31.85 ± 2.72 ^c^
	C-T3-50S+50DL	16	27.90 ± 3.19 ^b^	33.10 ± 3.57 ^b^	35.45 ± 3.80 ^b^
	C-T4-50S+50RS	56	32.55 ± 3.35 ^a^	41.15 ± 4.27 ^a^	44.10 ± 4.64 ^a^
	C-T5-50S+50SL	24	27.80 ± 0.70 ^b^	33.45 ± 2.06 ^b^	35.85 ± 2.50 ^b^
	C-T6-50S+50TL	16	25.20 ± 1.06 ^c^	32.80 ± 2.04 ^b^	35.55 ± 2.52 ^b^
*P. pulmonarius*	D-T1-100S	10	27.85 ± 2.43 ^cd^	36.00 ± 4.55 ^bc^	38.95 ± 5.27 ^bc^
	D-T2-50S+50CS	8	25.10 ± 2.47 ^e^	31.10 ± 3.19 ^d^	33.70 ± 3.66 ^d^
	D-T3-50S+50DL	18	29.65 ± 1.35 ^b^	38.20 ± 4.60 ^b^	41.15 ± 5.14 ^b^
	D-T4-50S+50RS	52	38.90 ± 2.47 ^a^	45.95 ± 3.61 ^a^	48.65 ± 4.17 ^a^
	D-T5-50S+50SL	18	26.95 ± 1.23 ^d^	33.75 ± 2.81 ^c^	36.50 ± 3.41 ^cd^
	D-T6-50S+50TL	14	28.85 ± 1.31 ^bc^	35.45 ± 4.58 ^c^	37.95 ± 5.12 ^c^

**Note:** Values are presented as mean ± standard deviation. Means within the same mushroom species followed by different letters indicate significant differences according to Duncan’s multiple range test (*p* < 0.05) compared with the control. Statistical comparisons were performed only among treatments within the same species; therefore, overall mushroom yield was not directly compared between species. Treatment A refers to *L. sajor-caju* cultivation, Treatment B refers to *P. cornucopiae* cultivation, Treatment C refers to *P. ostreatus* cultivation, and Treatment D refers to *P. pulmonarius* cultivation.

**Table 4:** Overall mushroom yield and biological efficiency of *Lentinus* and *Pleurotus* species cultivated on different substrate formulations.

Mushroom Species	Treatment	Number of Flushes	Number of Fruiting Bodies	Total Fresh Weight Per Bag (g)	Total Dried Weight Per Bag (g)	BE (%)
*L. sajor-caju*	A-T1-100S	2.09 ± 1.06 ^a^	14.59 ± 7.11 ^a^	54.54 ± 34.34 ^a^	6.10 ± 3.37 ^ab^	44.89 ± 18.85 ^b^
	A-T2-50S+50CS	2.55 ± 1.26 ^a^	16.64 ± 7.44 ^a^	70.75 ± 28.85 ^a^	8.09 ± 3.14 ^a^	61.99 ± 14.01 ^a^
	A-T3-50S+50DL	1.23 ± 1.11 ^b^	7.50 ± 6.38 ^b^	30.36 ± 28.11 ^b^	3.33 ± 2.89 ^c^	30.20 ± 10.83 ^c^
	A-T4-50S+50RS	1.32 ± 0.89 ^b^	6.68 ± 4.80 ^b^	26.71 ± 20.36 ^b^	3.39 ± 2.38 ^c^	17.70 ± 9.03 ^c^
	A-T5-50S+50SL	2.18 ± 0.91 ^a^	15.77 ± 7.32 ^a^	65.14 ± 28.41 ^a^	6.58 ± 2.57 ^ab^	56.54 ± 19.41 ^ab^
	A-T6-50S+50TL	1.86 ± 1.70 ^ab^	10.23 ± 9.38 ^b^	33.07 ± 30.68 ^b^	5.76 ± 5.50 ^b^	46.91 ± 14.41 ^b^
*P. cornucopiae*	B-T1-100S	2.29 ± 0.71 ^a^	15.83 ± 6.56 ^b^	90.34 ± 32.93 ^a^	ND	78.01 ± 5.21 ^c^
	B-T2-50S+50CS	2.14 ± 0.69 ^a^	16.60 ± 6.67 ^ab^	90.57 ± 38.86 ^a^	ND	102.79 ± 7.24 ^a^
	B-T3-50S+50DL	1.97 ± 1.04 ^a^	11.94 ± 7.13 ^c^	67.07 ± 39.28 ^bc^	ND	73.64 ± 7.50 ^d^
	B-T4-50S+50RS	1.00 ± 0.91 ^b^	7.91 ± 7.74 ^d^	50.48 ± 45.73 ^c^	ND	66.52 ± 4.17 ^e^
	B-T5-50S+50SL	1.97 ± 0.82 ^a^	20.01 ± 8.53 ^a^	89.02 ± 48.00 ^a^	ND	86.58 ± 3.04 ^b^
	B-T6-50S+50TL	2.11 ± 0.80 ^a^	13.94 ± 6.80 ^bc^	83.83 ± 45.64 ^ab^	ND	87.35 ± 4.55 ^b^
*P. ostreatus*	C-T1-100S	2.06 ± 0.76 ^a^	19.43 ± 9.86 ^a^	77.33 ± 36.26 ^bc^	ND	55.73 ± 2.87 ^c^
	C-T2-50S+50CS	2.11 ± 0.72 ^a^	21.31 ± 10.89 ^a^	93.74 ± 29.06 ^ab^	ND	99.40 ± 2.59 ^a^
	C-T3-50S+50DL	2.20 ± 0.87 ^a^	21.51 ± 11.43 ^a^	78.59 ± 37.99 ^bc^	ND	55.25 ± 1.67 ^c^
	C-T4-50S+50RS	1.43 ± 1.09 ^b^	12.09 ± 10.26 ^b^	52.77 ± 42.55 ^d^	ND	54.87 ± 1.64 ^c^
	C-T5-50S+50SL	2.00 ± 0.64 ^a^	22.31 ± 10.31 ^a^	97.43 ± 36.84 ^a^	ND	86.27 ± 2.12 ^b^
	C-T6-50S+50TL	2.06 ± 0.68 ^a^	23.43 ± 12.05 ^a^	73.00 ± 40.77 ^c^	ND	46.47 ± 1.50 ^d^
*P. pulmonarius*	D-T1-100S	2.03 ± 0.79 ^a^	8.54 ± 4.59 ^abc^	77.49 ± 40.21 ^bc^	ND	58.72 ± 6.64 ^d^
	D-T2-50S+50CS	2.29 ± 0.67 ^a^	11.06 ± 5.81 a	106.75 ± 45.29 ^a^	ND	101.96 ± 5.80 ^a^
	D-T3-50S+50DL	1.86 ± 0.77 ^ab^	8.23 ± 4.83 ^bc^	71.654 ± 40.97 ^bc^	ND	47.17 ± 1.60 ^e^
	D-T4-50S+50RS	1.57 ± 1.22 ^b^	7.46 ± 5.93 ^c^	63.27 ± 56.23 ^c^	ND	73.63 ± 2.30 ^c^
	D-T5-50S+50SL	2.26 ± 0.82 ^a^	10.00 ± 5.00 ^abc^	93.62 ± 45.47 ^ab^	ND	97.81 ± 3.26 ^b^
	D-T6-50S+50TL	2.26 ± 0.78 ^a^	10.71 ± 5.41 ^ab^	88.70 ± 41.95 ^ab^	ND	47.40± 1.35 ^e^

**Note:** Values are presented as mean ± standard deviation. Within each mushroom species, means followed by different letters indicate statistically significant differences according to Duncan’s multiple range test (*p* < 0.05) compared with the control. Statistical comparisons were performed only among treatments within the same species; therefore, overall mushroom yield was not directly compared between species. Treatment A refers to *L. sajor-caju* cultivation, Treatment B refers to *P. cornucopiae* cultivation, Treatment C refers to *P. ostreatus* cultivation, and Treatment D refers to *P. pulmonarius* cultivation. ND = data not recorded due to limitations in farm-scale facilities.

**Table 5:** Proximate composition (%) and energy values (kcal/100 g) of dried *Lentinus* and *Pleurotus* fruiting bodies cultivated on different substrate formulations.

Mushroom Species	Treatment	Dry Mass (%)	Moisture (%)	Ash (%)	Carbohydrates (%)	Energy Value (kcal/100 g)	Fat (%)	Fiber (%)	Protein (%)
*L. sajor-caju*	A-T1-100S	90.92	9.08	4.37	35.81	380.95	1.77	29.32	19.66
	A-T2-50S+50CS	90.92	9.08	4.43	37.70	380.81	1.26	26.90	20.63
	A-T3-50S+50DL	90.86	9.14	4.78	36.46	384.89	1.55	27.91	20.16
	A-T4-50S+50RS	90.65	9.35	4.30	35.75	394.48	1.62	29.98	19.00
	A-T5-50S+50SL	90.97	9.03	4.20	36.52	381.69	1.45	30.58	18.22
	A-T6-50S+50TL	90.27	9.73	4.50	35.44	388.48	1.55	27.17	21.63
*P. cornucopiae*	B-T1-100S	93.76	6.24	6.68	57.54	395.03	1.74	14.16	20.72
	B-T2-50S+50CS	93.09	6.91	5.84	54.07	388.49	1.35	13.91	24.87
	B-T3-50S+50DL	92.18	7.82	7.42	45.14	394.90	1.64	13.22	31.37
	B-T4-50S+50RS	94.15	5.85	6.51	53.80	384.82	1.62	13.22	25.62
	B-T5-50S+50SL	92.17	7.83	5.50	56.03	388.04	1.65	13.47	22.25
	B-T6-50S+50TL	93.17	6.83	8.26	51.96	387.03	1.95	13.77	24.11
*P. ostreatus*	C-T1-100S	93.64	6.36	6.78	54.40	402.80	1.57	12.34	24.71
	C-T2-50S+50CS	93.38	6.62	6.71	48.35	405.00	1.72	12.12	21.61
	C-T3-50S+50DL	93.20	6.80	6.54	48.68	403.09	1.46	11.96	30.53
	C-T4-50S+50RS	93.73	6.27	6.26	57.80	399.57	1.48	14.86	20.77
	C-T5-50S+50SL	92.99	7.01	6.32	57.19	385.38	1.67	13.16	21.23
	C-T6-50S+50TL	94.78	5.22	7.42	54.86	391.06	1.66	24.47	18.61
*P. pulmonarius*	D-T1-100S	94.19	5.81	6.40	56.19	389.63	1.34	10.26	25.13
	D-T2-50S+50CS	94.30	5.70	5.83	57.79	387.36	1.31	11.23	23.77
	D-T3-50S+50DL	94.25	5.75	6.95	57.81	395.36	1.33	10.29	23.01
	D-T4-50S+50RS	94.63	5.37	5.77	60.24	397.22	1.16	9.21	22.86
	D-T5-50S+50SL	93.98	6.02	5.56	57.32	381.40	1.11	12.47	23.76
	D-T6-50S+50TL	94.45	5.55	6.73	56.51	390.55	1.55	8.46	25.43

**Note:** Values are presented as proximate composition on a dry-weight basis.

**Table 6:** Comparative analysis of estimated substrate production costs under different farm scales and annual production capacities using 50% biomass replacement.

Farm Scale and Annual Production (Bags/Year)	Sawdust Required (Tons)	Sawdust Cost (100% Use, USD)	Sawdust Cost (50% Use, USD)	Biomass Required (50% Replacement, Tons)	Biomass Cost (USD)	Total Substrate Cost (50:50, USD)
Small scale =1000–30,000	0.6–16.8	50–1400	25–700	0.3–8.4	10–280	35–980
Medium scale =30,001–200,000	16.9–112.0	1408–9333	704–4667	8.5–56.0	283–1867	987–6533
Large scale =200,001–600,000	112.1–336.0	9342–28,000	4671–14,000	56.1–168.0	1870–5600	6541–19,600
Industrial scale ≥600,000	>336.1	>28,008	>14,004	>168.1	>5603	>19,608

**Note:** Production costs were estimated based on sawdust and biomass prices of USD 83.33 and 33.33 per ton, respectively. The 50:50 substrate formulation consisted of 50% sawdust and 50% agricultural biomass (*w*/*w*).

## Data Availability

The original contributions presented in this study are included in the article. Further inquiries can be directed to the corresponding authors.
